# On the Employment of Taxis in Strangulated Hernia

**Published:** 1872-02

**Authors:** C. C. F. Gay


### ART. III.—*On the employment of Taxis in Strangulated Hernia.*

By C. C. F. Gay, M. D.

Mr. President and Gentlemen:

Having observed the progress of several cases of strangulated hernia, during the past eighteen months, in which taxis was successfully employed, I have thought the observation of sufficient value to the profession to make record of them. Whenever a hernia, that is strangulated, is spoken or thought of, there is almost always, I think, associated therewith, as necessary to its cure, the use of the knife. But it should be borne in mind, that there are degrees of strangulation. One case of strangulation may be so slight in degree, as to produce no serious mischief, although it may have existed for several days without reduction: while another may exist that will cause the death of a patient in from eight to twelve hours, unless relief be obtained at once, since the inflamation would be so active and acute, especially if the patient be plethoric, that the death of the part would be inevitable, unless the knife be used promptly and early.

Therefore, in treating strangulated hernia, whether mild or severe in degree, the first thought that will suggest itself to the surgeon in the application of the taxis, will have reference to the *time* that he will be warranted in consuming in his efforts at reduction. The time consumned in the use of taxis will of course depend upon several circumstances. If the hernia be recent, the surgeon will expect to find a more dangerous condition than he would find had the hernia been old, since in recent hernia it must be presumed that it had been neglected, perhaps through ignorance of the patient, until the part had become acutely inflamed; so much so, indeed, as to preclude the possibility of prolonged taxis, since the slightest manipulatious would perhaps be inconsistent with the safety or healthfulness of the protruding and inflamed bowel. Another circumstance existing to determine the time that the surgeon may occupy in his efforts at the reduction of hernia, would be the general condition of his patient. He would examine the frequency and character of the pulse, ascertain the amount of febrile re-action—and the state of the patients stomach. If there were no stercoraceous vomiting or other alarming symptoms, although the parts protruding were extremely tender to the touch, an effort of from eight to ten minutes would be all the time a surgeon would be warranted in consuming. If not successful at the expiration of this time he should desist from further present efforts, and make use of the local application of ice, and continue its use interruptedly for three or four hours should he find it necessary, or what would be better perhaps before applying ice, deplete the inflamed part by the use of half dozen leeches, and afterwards make use of the ice. At the expiration of a few hours, or an hour perhaps, he might be able to return to his patient and reduce the hernia in a moment.

But, on the other hand should the symptoms be more urgent, the pulse rapid and the ejecta from the stomach be of a stercoraceous character, prompt action would be required and the knife used at once.

In case of an old hernia, since the inflammation would be less acute, the patient could be left several days with entire safety, if in the supine position and with ice or some refrigerating lotion, or perhaps better still,fomentations with cloths wrung out of hot water, applied over the inflamed parts, but of course the duration of the time when safety would cease and danger begin must depend upon the general symptoms, together with the amount of local inflammation present. I shall be justified in stating that the judicious surgeon will never operate simply because the hernia is strangulated. The majority of cases may be relieved without incurring the danger of an operation. Although aware, as I am, that this statement conflicts with the views of authors and the teachings of the schools, I think I shall be able to refer to cases that have occurred in my own practice, and observed in the practice of medical friends with whom I have been associated, illustrative of, and pointing clearly to the truthfulness of what I herein assert. No infallible rule can be laid down having reference to the manner of applying the taxis or governing the position of the patient. The rules heretofore suggested are entirely too arbitrary. The management of any single case must stand upon its own individual merits. He who possesses most tact will best succeed. He who has no tact—and there may be such—had better never try to reduce a hernia. Nothing better than the rules laid down by authors—if I except views that are arbitrary—can be suggested that I am aware of in reference to the method of employing taxis. I might, however, be allowed simply to suggest that often it would be wiser in grasping a hernial tumor for the purpose of its reduction, to pull upon it rather than push upon it, in other words while making gentle pressure over and around the tumor with the hand, if the tumor be of sufficient size to be grasped by the hand, gently drawing away the contents from the point of stricture, so as to diminish the relative size of the protruding bowel near its exit at the abdominal ring. The late lamented Dr. O. C. Gibbs, published an article January, 1869, in which he says : "I have been in practice twenty years and have never been compelled to use the knife for the relief of strangulated hernia." The plan practiced by Dr. Gibbs was the same as that recommended by Dr. Seutin, a Brazilian Surgeon, viz : "Rupture of the stricture by introducing the index finger through the stricture and using considerable force in stretching and lacerating it. If this method could be made available and so supercede the necessity of any further study of the anatomical relations of the parts involved, and of any further use of the knife, it would certainly be an advance in conservative surgery that would redound to the credit of its author. I think it worthy of trial as auxillary to the taxis, yet am quite skeptical as to the utility or safety of the procedure. Might there not be greater probability of lacerating the bowel than rupturing the structure?

The remark, however, made by Dr. Gibbs, as to the length of time he had practised his profession without the use of the knife in strangulated hernia, is significant. This remark, coming from so wise and skillful a surgeon as was Dr. Gibbs, should exert its influence for good, as I doubt not it will, by way of deterring from a too precipitate haste, in the employment of so dangerous an expedient as the use of the knife.

On January 11th, 1871, I was requested by Dr. Bartlett of this city to visit a patient with him residing on Tenth Street, and to go prepared to operate for strangulated femoral hernia. I found a patient 84 years old, with frequent pulse, who had been vomiting stercoraceous matter. The hernia was supposed to have been strangulated four days. It was recent, small, and upon the left side. There had been much local inflammation, but with appropriate topical application this had been somewhat allayed. Dr. Bartlett and myself both agreed that either with or without operation the old lady could live but a few days, and therefore advised against an operation, leaving the responsibility of choosing or electing to the friends of the patient, saying to them, that, should they request me to operate, I would do so, but only upon condition of such request.

Dr. BARTLETT has kindly furnished me with the subsequent history and happy termination of the case. I should add that the protrusion was probably omental.

The *position* of the patient is worthy of consideration in all cases where the taxis is employed. In this regard, authors who have heretofore taught that the supine posture, either upon a hard mattress or the floor with the hips elevated, or that the patient should at times be turned topsy-turvy, these being positions prerequisite to success, have failed, I think to teach the whole truth, as experience and observation demonstrates. There are other positions of the body equally essential to success as those above enumerated. The upright position is one of them, and the semi-prone position is another. I have succeeded in reducing hernia after I had failed with the patient placed in all other positions (save the upright) by placing the patient upon the side of the hernia in the semi-prone position, with the thigh flexed upon the body. I have in this position reduced a hernia almost instantly, after long trial in other positions. I have never yet succeeded by turning the patient topsy-turvy in reducing hernia. I once resorted to this method for experiment, after an operation. The stricture I had divided with the knife, there seemed no obstacle in the way of the return of the bowel, but the bowel did not return even when the patient was placed almost in a vertical position, with the head down. I have twice or thrice, after the patient's system had been well relaxed, taken the patient by the legs, after the manner taught in the books, and dragged him across the room with his legs over my shoulders, and his head dragging upon the floor, and have exhausted my strength in this way to no purpose. Never in a single instance have I met with success by this manœuvre. It is not strange that those who have commenced practice with the belief that such aposition is proper, should be slow to place any faith in the efficiency of the apposite or upright position. I have a most interesting and instructive case in point, recorded in my note book that I will briefly relate. I was called to this case in consultation by Prof. Wetmore.

Mrs. P., aged 42 years had femoral hernia of right side, of one year duration, wears a truss. When the truss was left off, the bowel protruded and could not be returned by persistent efforts of Dr. Wetmore, while the patient was under the influence of chloroform. This was late in the evening, local applications were made use of, and the next morning I visited the patient along with the doctor; chloroform was again administered when both of us failed to reduce the hernia after somewhat prolonged trial, with patient in supine position. I advised an operation, and during the absence of Dr. W. for chloroform and an assistant I entered into conversation with the patient upon her own mode of reduction of her hernia, when she stated that she always, before this, had had no difficulty in reducing it when she was standing upright. I said to her that she should have made this statement before, and requested her to stand up, when upon slight pressure over the tumor I felt a gurgling sensation, that was evidence of the return of the tumor, and she stated that she felt it returning. Convinced that the hernia could now be reduced, but not wishing to accomplish it in the absence of Dr. Wetmore, as no detriment could accrue to the patient, I requested her to lie down again. As soon as the doctor returned she again got up, placing her hand upon the tumor it was immediately reduced by herself. In order to make the report of this case a little nearer perfect, I should state that the patient, while in the upright position, had a tendency to syncope, and this circumstance undoubtedly facilitated the reduction of the hernia, and perhaps was as essential an element toward its accomplishment as the position itself, yet the fact remains that the patient often had occasion to reduce her own hernia, and never could succeed in any other position than the upright one· Whether she always had a tendency to faintness during her manipulations is not known.

During the past summer I reduced a femoral hernia, right side, by placing the patient upon her right side, nearly in the semi-prone position, with her thighs flexed upon the body, seizing hold of the tumor I almost immediately reduced it without the aid of chloroform, when I had failed with the patient in almost all other positions when she was under the use of chloroform. I am to conclude therefore that the positions heretofore recommended by the books are not always the best positions, and that if failures occur in such positions, then it will be wise to resort to the upright and if need be the semi-prone position, when taxis applied will be made most serviceable and efficient.

I am quite willing now to advance a step and claim that to reduce hernia, whether inguinal or femoral, by taxis, that the semi-prone or upright positions of the patient are always advisable, and I am quite willing to use stronger language, and assert, that the two positions named are the best, and the supine posture the worst for the patient to assume. It is not relaxation, but expansion of the abdominal parietes that we want.

But yesterday I reduced an inguinal hernia while my patient was standing, after I had made an ineffectual attempt at reduction with my patient lying upon his back.

On the evening of December 29th, 1871, I visited along with Drs. Diehl and Daggett, a female aged 73 years. I was requested by her attendant, Dr. Diehl, to go prepared to operate for strangulated femoral hernia. I found the patient with frequent pulse, learned that her hernia had been strangulated, and that she had been vomiting for twenty-four hours, and the daughter stated that what her mother vomited was "bad smelling." She was lying with her head elevated and legs flexed. The hernia was upon the left side, about the size of a black walnut and tender to the touch. Trial had been made during the day to reduce it by taxis, and another trial was now made, the woman in the position above described. The tumor felt tense and was hard and unyielding. Immediately I became convinced that no one could reduce the hernia while the patient remained in this posture, therefore I at once turned her over upon the side of the hernial protrusion, placing her in the semi-prone position, and with the fingers of my left hand reduced the hernia with the greatest ease, not occupying more than two minutes of time. She made a good recovery notwithstanding the existence of strangulation for twenty-four hours.

Hernial protusion always occurs when the person is standing. In this position it is easier far, for the bowel, or a portion of the contents of the abdominal cavity, to pass through an open space; why then, not return the protruding bowel, with the patient occupying precisely the same attitude that was occupied when a portion of bowel emerged from the cavity through the outlet or ring? I am not really conscious that position exerts any considerable influence over stricture, still I am inclined to the belief that it does in some way or another modify the intensity of a stricture. An opening, made through a hollow rubber ball, or a hole made into any spheroidal hollow flexible body of any material, would be invisible when such body or substance was in a state of partial or complete collapse, but expand or inflate the ball, and the opening or hole through it will likewise expand proportionately, so that what before seemed to be a mere puncture is now a good sized opening.

The abdomen may be likened to a ball or spherical body, the parieties of which are capable both of expansion and contraction or collapse. Standing; the weight of the abdominal viscera is downwards, and the persons abdomen may be said to be in a state of expansion. Lying down; in the supine posture the downward pressure of the viscera is taken off and the abdomen is in a condition of contraction or collapse.

This explanation or rather illustration is merely a mechanical one, and may carry no particle of conviction along with it, yet I cannot but think that conclusions herein suggested, would be verified by experiments upon the cadaver, which would convert mechanical illustration into anatomical demonstration. If there be any foundation in fact, for this new theory to rest upon, for the relief of stricture, then am I justifiable in asserting, that for the most successful and efficient application of the taxis in strangulated hernia, whether inguinal or femoral, the patient should be required to stand upon his or her feet, or to assume the semi-prome position, rather than to be upon the back.

Since the above was written I have received from the publishers a copy of the "The Georgia Medical Companion." The November number contains an article entitled. "Reduction of Hernia in the Erect Posture," copied from the Canada Lancet, by Dr. McGeachy. It is the report of a case in which the patient was relieved by taxis in the erect posture.

For the purpose of embodying in this paper the literature of the most recent date, touching the subject matter of which I write, I will copy the concluding two paragraphs of Dr. McG.'s article. He says: "I believe that the proper position, theoretically, for the reduction of a strangulated inguinal hernia, and in which alone the co-operation of dynamic agencies can be utilized, is the erect posture, with the flexure and adduction of the thigh.

The means to be used are obvious. If beforehand the colon be well evacuated, or as much so as possible, every rational preparatory condition will have been fulfilled.

In the old positions, but one force is brought to bear—the *pushing force*, used by the operator, if I may so term it. By this method we have also a pulling force, (*vis a fronte,*) namely, the weight of a large portion of the bowel striving to drag the remainder from its posture of imprisonment. Why not, then, invert the patient and secure the action of this new force in a still greater degree? Simply this: The rhythmic action of the diaphragm forbids the continued operation of this force, and should it have any effect, it often leaves matters *in statu quo* during its contraction. Besides the force here would generally be acting at an angle the ring being the fixed point."

I will conclude this paper by stating a fact that should be taken into account, when considering the best posture of a patient upon whom taxis is to be employed, a fact too, that has hitherto been overlooked by writers upon this subject. I allude to the fact that all or nearly all persons afflicted with hernia never get down upon their backs, but on the contrary stand upright and reduce their own hernia's. I shall only add that I shall henceforth believe, and act upon such belief until convicted of error, that the semi-prone and upright postures have a tendency to, if they do not absolutely, dilate the structure. If this belief has foundation in fact, then the use of taxis will supercede the necessity of the use of the knife in a majority, if not in all the cases of strangulation that may occur.*

* Read at the regular meeting of the Medical Association.

